# A Systematic Review of Graft‐Related Complications and Recurrence Following Minimally Invasive Sacrocolpopexy With Xenografts and Allografts

**DOI:** 10.1111/1471-0528.70184

**Published:** 2026-03-02

**Authors:** Marije A. Boom, Margot van Genderen, Paul M. Verheijen, Esther C. J. Consten, Henk W. R. Schreuder, Steven E. Schraffordt Koops

**Affiliations:** ^1^ Department of Surgery UMC Groningen Groningen the Netherlands; ^2^ Department of Surgery Meander Medical Centre Amersfoort the Netherlands; ^3^ Department of Gynaecology and Obstetrics Meander Medical Centre Amersfoort the Netherlands; ^4^ Department of Gynaecologic Oncology UMC Utrecht Utrecht the Netherlands

**Keywords:** biological, erosion, exposure, GRC, meta‐analysis, pelvic organ prolapse, recurrence, sacrocolpopexy, systematic review

## Abstract

**Background:**

Biological grafts are proposed as an alternative to synthetic grafts in sacrocolpopexy (SC) to reduce complications such as graft exposure and immunologic reactions. However, concerns remain long‐term durability. This systematic review and meta‐analysis assess recurrence rates and graft‐related complications (GRC) in minimally invasive sacrocolpopexy (MISC) using biological grafts.

**Objectives:**

To evaluate the recurrence and GRC following MISC with biological grafts and to compare outcomes across different materials.

**Search Strategy:**

PubMed, Embase and Cochrane databases were searched up to August 2024.

**Selection Criteria:**

Studies with ≥ 10 patients undergoing MISC with biological grafts, reporting recurrence or GRC with ≥ 6 months follow‐up, were included.

**Data Collection and Analysis:**

Two independent reviewers assessed study quality using the Newcastle‐Ottawa Scale (NOS). A random‐effects meta‐analysis estimated pooled recurrence and GRC with 95% confidence intervals (CI).

**Main Results:**

Five studies (353 patients) met inclusion criteria. Recurrence rates ranged from 2.4% to 52.6% (pooled: 25.6% [95% CI: 8.1%–48.2%]). Graft exposure occurred in 0.8% (95% CI: 0.0%–4.2%). ADM had the lowest recurrence (2.4%) with no reported exposures, whereas Tutoplast cadaveric fascia lata showed the highest recurrence (52.6%) and exposure (5.3%). The pooled reoperation rate for recurrence was 16.8% (95% CI: 0.0%–51.7%).

**Conclusion:**

This systematic review identified a higher recurrence rate with biological grafts in MISC than typically reported for synthetic grafts in other literature, while exposure rates appeared to be comparable. Given study heterogeneity, further research is required to determine the optimal graft choice balancing durability and complication risk.

**Abbreviations**
ADM: acellular dermal matrix
AS: absorbable sutures
CI: confidence interval
FDA: Food and Drug Administration
GRC: graft‐related complications
MISC: minimally invasive sacrocolpopexy
MUS: mid‐urethral sling
NOS: Newcastle‐Ottawa Scale
PDS: polydioxanone sutures
POP: pelvic organ prolapse
POPQ: pelvic organ prolapse quantification
PRISMA: Preferred Reporting Items for Systematic Reviews and Meta‐Analyses
PS: permanent sutures
SC: sacrocolpopexy
SIS: small intestine submucosa

## 1 Introduction

Pelvic organ prolapse (POP) is a significant health concern for women, with a prevalence of up to 50% in parous women and an estimated lifetime risk of surgical intervention ranging from approximately 11%–19%, [1, 2]. POP can lead to severe symptoms and significantly reduce the quality of life for affected individuals [3]. Sacrocolpopexy (SC) is a commonly performed surgical procedure for the management of POP, particularly favoured for correcting vaginal vault prolapse due to its superior anatomical and functional outcomes [4, 5]. This procedure can be performed through laparotomy or minimally invasive approaches (laparoscopic or robotic) and requires the use of an implant to correct the deficiency in level I support [6]. While synthetic grafts have traditionally been favoured for their excellent long‐term anatomical results, concerns have arisen due to the emergence of complications, notably graft exposures and pain, prompting the exploration of alternative options [7, 8]. The U.S. Food and Drug Administration (FDA) warnings in 2008 and 2011 specifically addressed permanent synthetic meshes used in transvaginal prolapse repair, where high rates of exposure, pain, and dyspareunia were reported [9]. These warnings did not apply to abdominal approaches such as SC, where synthetic mesh continues to be widely used due to its superior durability. Nevertheless, even in SC, mesh exposure and chronic pain remain complications of concern, underscoring the search for safer alternatives such as biologic grafts [10].

Permanent synthetic materials tend to elicit an inflammatory response, which may account for the longevity of repairs but also contributes to graft‐related complications (GRCs) [11]. The density of the implant material influences the degree of inflammation [11]. Consequently, lightweight polypropylene (PP), allografts, and xenografts have been introduced, provoking milder reactions [12]. Xenografts, serving as a collagen scaffold for soft tissue remodelling and regeneration of native tissue, may reduce GRC by avoiding the use of permanent foreign material. However, long‐term durability remains uncertain and is an ongoing area of research.

This systematic review and meta‐analysis aimed to establish an overview of the currently available literature regarding minimally invasive sacrocolpopexy (MISC) to determine the incidence of GRC and recurrences after utilising biological grafts. As evidence to date is limited to small and heterogeneous studies, this review provides the first pooled analysis of biologic graft use in MISC, addressing an important gap in the literature.

## 2 Materials and Methods

This systematic review was conducted and reported following the Preferred Reporting Items for Systematic Reviews and Meta‐Analyses (PRISMA) guidelines [13]. Search strategies, eligibility criteria, the used critical appraisal tool, and outcomes of interest were pre‐specified. The study was prospectively registered in PROSPERO (study ID: CRD42024577997).

### 2.1 Eligibility Criteria

To gain an overview of GRCs associated with biological grafts in MISC, studies were considered eligible if they met the following criteria: (1) included patients who underwent SC or sacrohysteropexy with a heterologous biological graft/mesh; (2) employed a minimally invasive surgical technique; (3) reported on recurrence or GRCs as primary or secondary outcomes; (4) included at least 10 patients; (5) had a follow‐up period of at least 6 months; and (6) were observational studies (cohort studies, case–control studies) or clinical trials.

Studies were excluded if they: (1) were not published in English; (2) provided insufficient data on GRCs; (3) did not specify the type of graft used; (4) studied using autologous fascia; or (5) exclusively included studies that included patients undergoing concomitant surgical interventions, such as sacrocolporectopexy or mid‐urethral sling (MUS), without separately reporting outcomes for those who underwent MISC alone.

### 2.2 Search Strategy

The electronic databases of Pubmed, Embase and Cochrane were searched to identify relevant studies until August 2024. The literature search was conducted using the following terms: (sacrocolpopex*[tiab] OR sacral colpopex*[tiab] OR sacrocolpoperineopex*[tiab] OR colpopex*[tiab] OR colpoperineopex*[tiab]). There were no restrictions on the publication date. After excluding duplicate reports, two researchers (MAB and MAG) independently screened all studies based on title and abstract, followed by full‐text assessment of the selected studies. Finally, the reference lists of the eligible studies were reviewed to identify any additional relevant articles. Disagreement among the authors regarding the quality or relevancy of articles was resolved through discussion until consensus was reached.

### 2.3 Data Collection

Data were extracted from eligible studies using a standardised data form. The extracted data included study characteristics, patient demographics, prior surgery, types of (biological) grafts and sutures used, details of GRCs and recurrences. Prior surgery was defined as any previous prolapse repair in the anterior, posterior, or apical compartment, with or without concomitant hysterectomy. Incontinence procedures were generally reported separately and were not included under this definition. The outcome parameters of interest were GRCs and/or recurrence. GRCs were defined as the symptomatic or asymptomatic presence of graft exposure [12]. Recurrence was defined as postoperative POPQ score of ≥ 2 and/or the need for retreatment [14].

### 2.4 Risk of Bias

Two authors (MAB and MVG) independently assessed the methodological quality of each article utilising the Newcastle‐Ottawa Scale (NOS), which evaluates population selection, comparability, and outcome measurement [15]. The NOS ranges between zero up to nine stars, with a total score of six or higher indicating satisfactory quality. Randomised controlled trials were assessed separately using the Cochrane Risk of Bias 2.0 tool (RoB 2.0) [16].

### 2.5 Statistical Analysis

The proportional meta‐analysis was performed using the ‘meta’ package in R (version 2024.12.0+467). Plots and pooled incidences were generated directly in R to ensure transparency and reproducibility. Due to the frequent reporting of zero events for certain outcomes, an arcsine transformation was applied to calculate the overall incidence. Event data from individual studies were extracted and integrated using a random‐effects model. Following back‐transformation, the combined incidence proportions and their corresponding 95% confidence intervals (CI) were documented and visually depicted through forest plots. Statistical significance was determined at a threshold of *p* < 0.05. Heterogeneity was calculated using the *I* ^2^ statistics.

## 3 Results

**FIGURE 1:**
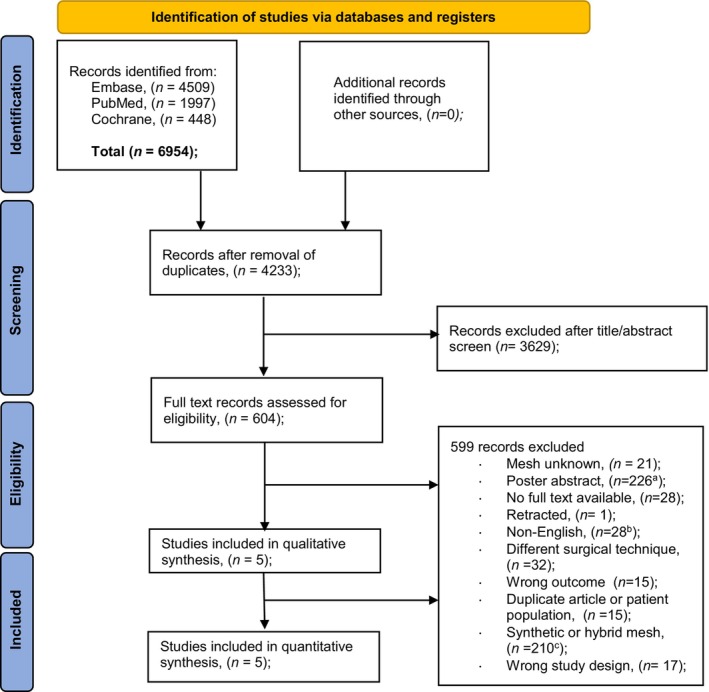
Preferred Reporting Items for Systematic Reviews and Meta‐Analyses study flow diagram showing selection of studies. ^a^Includes nine articles mentioning biological grafts and one mentioning a hybrid graft Ref. [17, 18, 19, 20, 21, 22, 23, 24, 25], [26]. ^b^Includes three articles mentioning biological grafts Ref. [27, 28, 29]. ^c^Includes six articles mentioning hybrid grafts Ref. [30, 31, 32, 33, 34, 35].

After database searches, 6954 articles were screened. Following the removal of duplicates, 4233 articles remained. Of these, 604 studies were deemed eligible for full‐text evaluation after title and abstract screening. Among these, 599 reports were excluded for various reasons. 21 reported unknown mesh types, 226 were conference poster abstracts, 28 lacked accessible full texts, 1 had been retracted, 1 was excluded due to overlapping patient groups, and 28 were published in non‐English languages. Additionally, 32 described alternative surgical techniques, 15 reported irrelevant outcomes, and 15 involved duplicate publications or overlapping cohorts. Synthetic or hybrid meshes (i.e., combined synthetic–biologic constructs were used in 210 articles, and 17 employed inappropriate study designs). Consequently, seven articles were considered eligible for inclusion. The authors of two studies were contacted to request further data, but no responses were received. This screening resulted in the inclusion of five articles (Figure 1) [36, 37, 38, 39, 40].

### 3.1 Quality Assessment

The NOS score results for each cohort study are shown in Table 1. Scores ranged from 4 to 8 out of 9. Two studies achieved a score of six or higher [37, 40], indicating satisfactory quality, whereas the remaining studies were rated below this threshold [38, 39]. Culligan et al. [36] was assessed separately using the RoB 2.0, as shown in Table 2, and was judged to have a low risk of bias (Tables 1 and 2).

**TABLE 1:** Newcastle‐Ottawa Scale (NOS) scores for cohort studies evaluating methodological quality.

	Selection				Comparability		Outcome recurrence			Outcome GRC			Total number of stars	
	1	2	3	4	1	2	1	2	3	1	2	3	Recurrence	GRC
Kerkhof et al. [38]	b★	0	a★	0	0	0	a★	a★	a★	a★	a★	a★	5	5
Loffeld et al. [36]	b★	a★	a★	0	0	0	a★	a★	b★	a★	a★	b★	6	6
Deprest et al. [39]	a★	a★	a★	a★	a★	0	a★	a★	0	a★	a★	0	8	8
Culligan et al. [35]	a★	a★	a★	a★	a★	0	a★	a★	b★	a★	a★	b★	8	8
Karon et al. [37]	b★	0	a★	0	0	0	0	a★	0	0	a★	0	4	4

*Note:* ★, one star; Selection: 1, adequate case definition; 2, representativeness of cases; 3, selection of controls; 4, definition of controls; Comparibility: 1, main factor, 2, additional factor; Outcome (recurrence or GRC) 1, ascertainment of exposure; 2, same method; 3, nonresponse rate.

Abbreviation: GRC, graft related complications.

**TABLE 2:** Risk of bias 2.0 tool (RoB 2.0) for randomised controlled trials.

	Randomisation process	Deviations from intended interventions	Missing outcome data	Measurement of outcome	Selection of reported result	Overall risk of bias
Culligan 2013	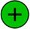	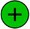	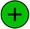	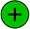	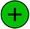	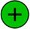

### 3.2 Study Characteristics

The baseline characteristics of the included studies are summarised in Table 3. A total of 353 patients were included across five studies [36, 37, 38, 39, 40]. Of these, 304 patients (86.1%) underwent laparoscopic SCP, while 49 patients (13.9%) underwent robotic‐assisted SCP. The studies were published between 2005 and 2019, with the median publication year being 2012 (range: 2005–2019). In terms of study design, two studies (40%) were prospective, while three studies (60%) were retrospective. All studies were conducted at single centres, with data primarily derived from medical records. Follow‐up durations ranged from 12 to 67 months, with four studies reporting a median or mean follow‐up exceeding 24 months [38, 40].

**TABLE 3:** Baseline characteristics.

	No. (% total)
Baseline characteristics	
Included studies	5
Patients	353
Laparoscopic SCP	304 (86.1)
Robotic SCP	49 (13.9)
Study characteristics	
Mid‐year (range)	2012 (2005–2019)
Design; prospective	2 (40.0)
Design; retrospective	3 (60.0)
Single center, medical records based	5 (100)
Follow‐up, mean (range)	32.4 (12–67.0) (months)
Patient characteristics (% total patients)	
Age, mean^a^	56.2
Prior prolapse surgery^a^	71 (of 107)^b^ (66.4)
Parity	2.8 (of 136)^b^

^a^ Not all studies reported on this outcome.

^b^ All patients of which this data was reported.

The mean age of patients across the studies was 56.2 years. Among 107 patients with available data on prior prolapse surgery, 71 patients (66.4%) had a history of prior pelvic surgery. Parity was reported for 136 patients, with a mean parity of 2.8.

### 3.3 Graft Related Complication

The included studies describe a total of 353 patients eligible for analysis of GRCs (Table 4). Of these, 211 patients (59.8%) were treated with ADM (non‐crosslinked acellular dermal matrix), 57 patients (16.1%) with porcine dermis (Pelvisoft), 50 patients (14.2%) with cross‐linked dermal collagen (Pelvicol) and SIS‐derived implants, and 35 patients (9.9%) with Tutoplast (dura mater, bovine pericard or cadaveric fascia lata). The sutures used for distal fixation of the mesh were either non‐resorbable or not specified.

**TABLE 4:** Recurrence incidence & graft exposure.

Author, year	Design^a^	*N*	Graft name^b^	Suture name	FU (months)	R/L^c^	Recurrence (%)	Anatomical recurrence (%) (POP‐Q/clinical assessment)	Retreatment (%) (reoperation for prolapse)	O/S^d^	Graft exposure (%)	Previous surgery (%)
Culligan [35]	P	57	Pelvisoft, (Porcine dermis, acellular collagen matrix)	PTFE (polytetrafluorethylene)	12	B	11/57 (19.3)	11/57 (19.3)	NR	O	1 (1.8)	21 (36.8)
Deprest [39]	P	50	Pelvicol (cross‐linked dermal collagen) & Surgisis (SIS derived implants)	NR	32.6 (20–68)	L	19/50 (38)	19/39 (49)	6/50 (12)	O	2 (4)	50 (100)
Karon [37]	R	211	ADM (now owned by Allergan, previously Life Cell)	GORE‐TEX sutures	45 (7–60)	L	5/211 (2.4)	NA	5/211 (2.4)	S	0 (0)	NR
Kerkhof [38]	R	16	Tutoplast: dura mater, fascia lata, bovine pericard	Ethibond sutures (Johnson & Johnson)	38.2	L	6/16 (37.5)	6/16 (37.5)	NR	O	0 (0)	NR
Loffeld [36]	R	19	Tutoplast (dehydrated cadaveric fascia lata mesh)	Non resorbable sutures (brand NR)	67	L	10/19 (52.6)	10/19 (52.6)	10/19 (52.6)	O	1 (5.3)	NR

^a^ P = Prospective design, R = Retrospective design.

^b^ Sutures used to attach mesh to vaginal vault.

^c^ R = robot (assisted) surgery, L = laparoscopic surgery, B = both.

^d^ O = Objective recurrence defined as evaluated at a clinical visit, or via the POP‐Q score. S = Subjective recurrence, defined as complaints of vaginal bulge.

Incidence of exposure varied across groups. In the ADM cohort, no exposures (0%) were observed after 12 months [38]. In the porcine dermis group, one graft exposure was reported (1.8%), which required surgical intervention involving partial excision of the exposed graft [36]. In the study involving cross‐linked dermal collagen (Pelvicol) and SIS‐derived implants, a 4% exposure incidence was reported, with all cases occurring in the Pelvicol group. These exposures were surgically managed, with partial removal of the affected graft. No exposures were reported for the SIS implants. Among the studies using Tutoplast, no exposures (0%) were reported in the dura mater group after a mean follow‐up of 38.2 months [39]. However, in the cadaveric fascia lata group, one exposure (5.3%) was observed at 67 months. This exposure was treated conservatively with topical oestrogen therapy [37].

**FIGURE 2:**
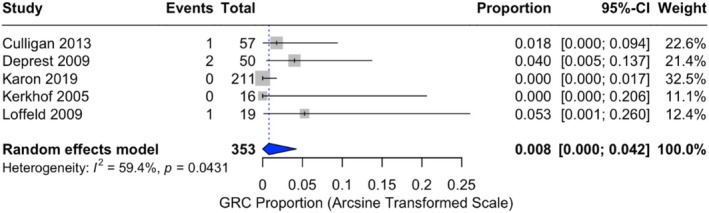
Forest plot of graft related complication proportions. Horizontal axis for arcsine of square proportion; Vertical axis for included studies in meta‐analyses. CI, confidence interval; GRC, graft related complications.

In total, four instances of exposure were reported across the included studies, yielding an overall proportion of 0.8% (95% CI: 0.0%–4.2%) with moderate heterogeneity observed (*I* ^2^ = 59.4%, *p* = 0.0431) (Figure 2). Subgroup analysis was not possible due to the small number of studies in each group.

### 3.4 Recurrence

Recurrence incidences were reported in all five studies [36, 37, 38, 39, 40], ranging from 2.4% to 52.6% with a pooled incidence of 25.6% (95% CI: 8.1%–48.2%) (Table 4). The lowest incidence of 2.4% was observed in 211 patients treated with an ADM graft after a mean follow‐up of 45 months [38]. Among 57 patients treated with porcine dermis grafts, a recurrence incidence of 19.3% was reported at 12 months [36]. For cross‐linked dermal collagen and SIS‐derived implants, the recurrence incidence was 38% at a mean follow‐up of 32.6 months [40].

**FIGURE 3:**
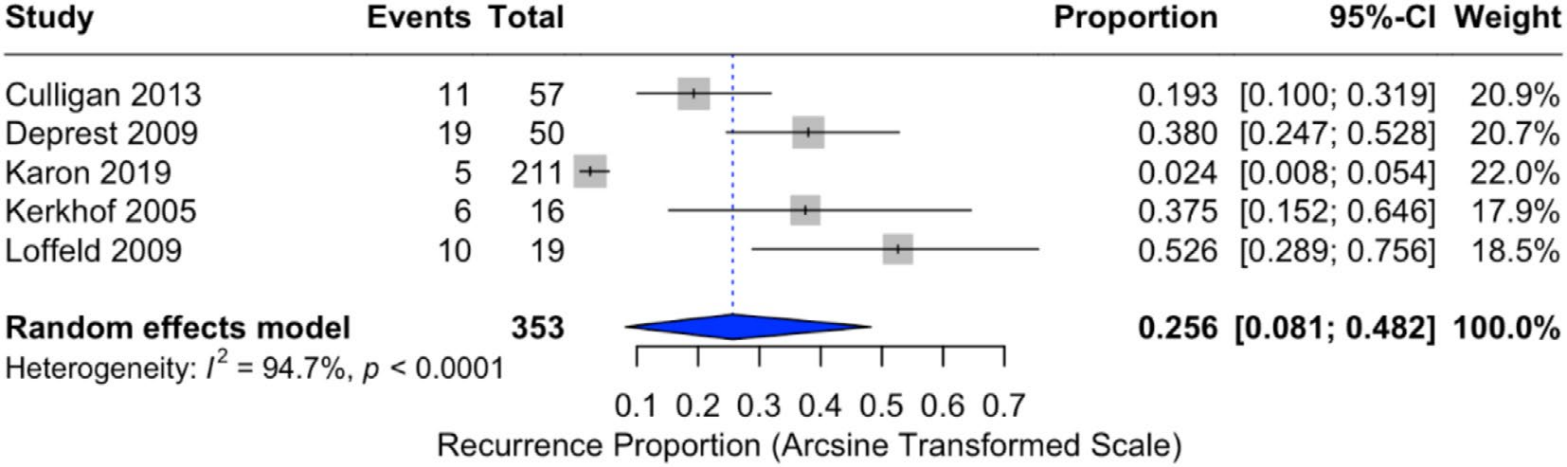
Forest plot of recurrence proportions. Horizontal axis for arcsine of square proportion; Vertical axis for included studies in meta‐analyses. CI, confidence interval.

Studies utilising Tutoplast grafts reported the highest risk of recurrence. Among 16 patients receiving a dura mater, fascia lata, or bovine pericardium graft, the incidence was 37.5% at a mean follow‐up of 38.2 months. For 19 patients treated with dehydrated cadaveric fascia lata grafts, the recurrence incidence was 52.6% at 67 months [37, 39]. There was strong evidence of statistical heterogeneity among these studies (*I* ^2^ = 94.7%, *p* < 0.0001) (Figure 3).

**FIGURE 4:**
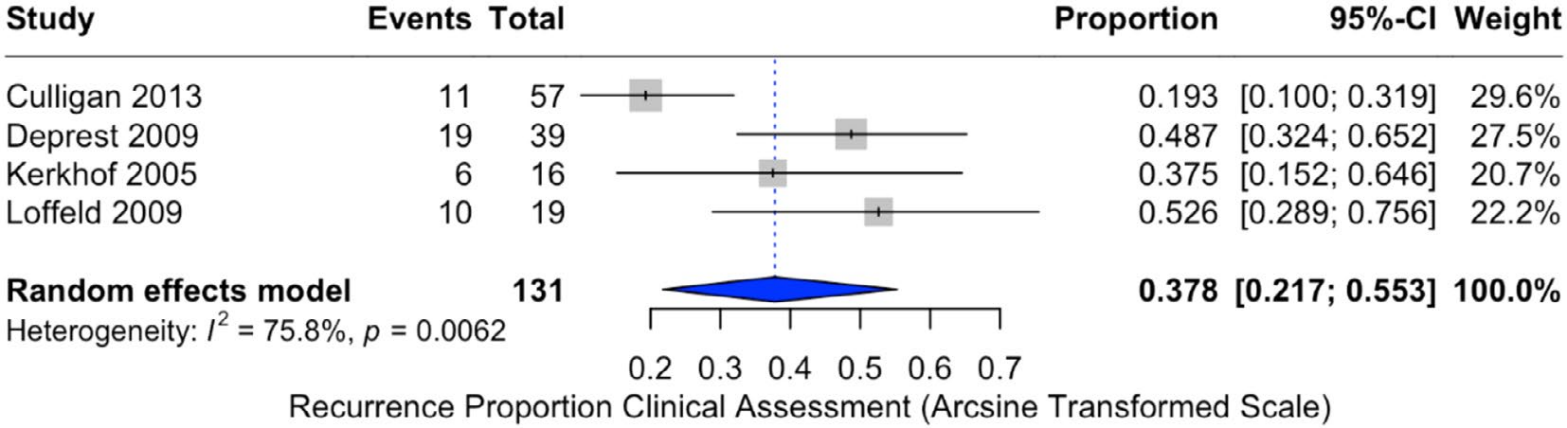
Forest plot of recurrence proportions with clinical assessment. Horizontal axis for arcsine of square proportion; Vertical axis for included studies in meta‐analyses. CI, confidence interval.

**FIGURE 5:**
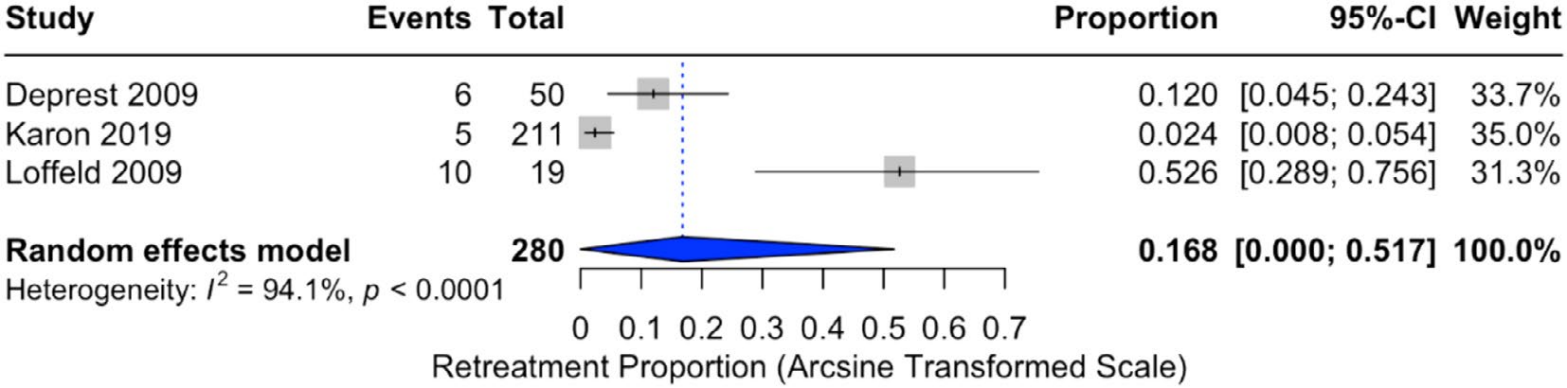
Forest plot of retreatment proportions. Horizontal axis for arcsine of square proportion; Vertical axis for included studies in meta‐analyses. CI, confidence interval.

When considering only patients with clinical assessment, the recurrence incidence was higher, with a pooled proportion of 37.8% (95% CI: 21.7%–55.3%). This analysis included 131 patients from four studies [36, 37, 39, 40] (Figure 4). Reoperations for recurrent prolapse were less frequent, with a pooled incidence of 16.8% (95% CI: 0.0%–51.7%), with significant heterogeneity (*I* ^2^ = 94.1%, *p* < 0.0001) (Figure 5).

## 4 Discussion

Robotic and laparoscopic SC are recognised as the gold standard for treating post‐hysterectomy vaginal vault prolapse, offering effective pelvic support through minimally invasive techniques. Synthetic grafts, known for their durability and high success rates, are widely used; however, their association with complications such as tissue damage, exposure, and extrusion has prompted exploration of alternative materials. Biological grafts, proposed to mitigate these complications, raise concerns about long‐term durability despite their theoretical benefits [5, 41]. While some studies examine biological grafts in gynaecological surgery, specific analyses of their role in MISC remain limited [42]. To our knowledge, this is the first systematic review and meta‐analysis specifically pooling outcomes of biological grafts in MISC.

This review identified a pooled recurrence proportion of 25.6% (95% CI: 8.1%–48.2.%) for biological grafts in MISC, with significant heterogeneity between studies (*I* ^2^ = 94.7%, *p* < 0.0001). Reoperations for recurrent prolapse were required in 16.8% of cases, underscoring the clinical burden of recurrence for patients and raising concerns about the long‐term durability of biological grafts. In contrast, the pooled incidence of graft exposure was estimated at 0.8% (95% CI: 0.0%–4.2%) under the random effects model, showing moderate heterogeneity (*I* ^2^ = 59.4%, *p* = 0.0431). The significant heterogeneity highlights variability in study design, graft materials, and patient populations. Smaller studies with higher recurrence and exposure proportions contributed more significantly under the random effects model, leading to higher pooled estimates.

Notably, when excluding the study by Karon et al. [38], the pooled recurrence proportion increased, while heterogeneity (*I* ^2^) decreased from 94.7% to 75.8%. This suggests that Karon's study contributed substantially to interstudy variability, likely due to methodological differences, including the absence of standardised anatomical follow‐up. The lower recurrence proportion reported may therefore reflect an underestimation due to the lack of clinical assessment.

Synthetic grafts have demonstrated superior long‐term outcomes in the literature, with a pooled success proportion of 93% and a reoperation risk of 3%, as reported across prospective studies [43]. Moreover, synthetic grafts maintain stable support over extended follow‐up, with a risk of exposure remaining below 10% even after 7 years [44]. However, when graft exposure occurs, synthetic meshes are more prone to chronic inflammation, infection, and erosion into adjacent structures, often necessitating complete removal [45, 46, 47]. This contrasts with biological meshes, which integrate with host tissue through revascularization and collagen remodelling, potentially allowing for conservative management in cases of exposure [48].

While this review focuses on MISC, evidence from open SC further supports these trends. Altman et al. [49] reported a 29% recurrence rate with porcine dermal xenografts, comparable to rates observed with minimally invasive techniques. Furthermore, Quiroz et al. [50] observed an 11% recurrence rate with Pelvicol, which was significantly higher than the 1% reported for synthetic grafts (*p* = 0.011) at a mean postoperative follow‐up of 1.1 ± 1.05 years. Despite these higher recurrence rates, the incidence of exposure with biological grafts remains consistently low across both open and minimally invasive techniques, ranging from 0% to 3% [49, 51]. This trend across surgical techniques highlights the challenge of balancing long‐term repair strength with the risk of complications.

The variability in the risk of recurrence and exposure among studies using biological grafts may be attributed to differences in graft materials. Biological grafts differ in origin, source, proprietary processing, cross‐linking, and sterilisation methods. Among the five studies included in this review, four different types of biological grafts were used, leading to significant variability in outcomes. Porcine‐derived tissues, including porcine dermis (Pelvicol, Pelvisoft) and small intestine submucosa (SIS, Surgisis), were used in three studies, while Tutoplast, a cadaveric human fascia, was used in two studies. These materials exhibit wide variability in strength, degradation timelines, and potential for host immune responses. For example, cross‐linked collagen grafts like Pelvicol may provide greater durability but carry a higher risk of inflammatory complications [46, 52]. Notably, in our results, two of the four reported exposures were associated with cross‐linked Pelvicol grafts, highlighting the potential inflammatory risks tied to specific processing techniques. Autologous fascia grafts, such as rectus fascia, fascia lata, and semitendinosus, were not included in this review. These grafts represent a distinct category, characterised by the use of patient‐derived tissue, associated donor‐site morbidity, and biomechanical and remodelling properties that differ fundamentally from those of heterologous biological grafts [10, 43, 48, 51].

The pursuit of an optimal repair material remains a key challenge in POP surgery. While biological grafts aim to mitigate complications such as exposure, their durability under prolonged pelvic stress is often limited. This has led to growing interest in combined synthetic–biologic constructs or hybrid grafts, which combine the durability of synthetic materials with the lower exposure risks of biological grafts [30, 31, 53]. Promising outcomes have been reported with combined synthetic–biologic constructs; for example, Shariati's robotic‐assisted approach using Prolene mesh in combination with porcine dermis reported an exposure risk around 4% and recurrence rate at 1% after one year [32]. Other studies found 0% exposure with hybrid grafts over two years and 18 months, with recurrence incidence ranging from 1% to 6.5% [33]. Skoczylas et al. [34] reported a 3% reoperation rate for mesh exposure in synthetic‐only grafts. In their study, no hybrid graft recipients required reoperation for exposure. They estimated that treating 33 patients with a hybrid graft could prevent one exposure, suggesting a potential reduction in reoperations and cost‐effectiveness. This represents a promising avenue for future investigation.

In addition to the variability in graft materials, differences in suture type may also impact surgical outcomes. In a recent meta‐analysis, Chen et al. [35] compared absorbable sutures (AS) and permanent sutures (PS) in SC. The study found no significant difference in the risk of graft exposure between the two groups (OR = 1.00; 95% CI, 0.49–2.08). However, AS demonstrated a significant reduction in suture‐related complications, including a lower risk of suture exposure/erosion (OR = 0.18; 95% CI, 0.06–0.58) and suture removal (OR = 0.14; 95% CI, 0.03–0.61), highlighting a potential safety advantage of absorbable materials. In this review, PS predominated, with all described sutures being permanent, or their permanence not specified. Notably, not all studies differentiated between graft exposure and suture exposure, which complicates the interpretation of suture‐related findings.

Several limitations need to be acknowledged in this review. Despite extensive screening of over 4200 articles, only five studies met our inclusion criteria, limiting generalizability. The small sample size and low incidence of graft exposures also reduced statistical power. In addition, this review represents a single‐arm meta‐analysis, pooling outcomes of biological grafts only. Therefore, no direct comparisons with synthetic grafts were possible, which further limits interpretation. Moreover, clinical heterogeneity—specifically in graft types and suture materials—further complicates interpretation. Finally, the absence of full‐text publications for several studies reporting on biological grafts [17, 18, 19, 20, 21, 22, 23, 24, 25, 26, 27, 28, 29], as indicated in the study selection process (Figure 1), suggests potential publication bias, limiting a comprehensive evaluation of their efficacy in SC.

Future research should prioritise larger, homogeneous studies comparing biological, synthetic and possibly hybrid grafts, with attention to both objective and subjective outcomes. Standardising graft types, suture materials, and techniques is crucial to reduce variability and improve reliability of results. Furthermore, the long‐term durability of biological grafts should be explored, particularly in specific patient populations at higher risk for complications.

## 5 Conclusion

While synthetic‐graft SC remains a widely used and effective reference procedure for apical compartment repair, with high long‐term anatomical success and generally low but persistent exposure rates, mesh‐related complications are clinically significant and must be considered during counselling. Emerging biological materials and hybrid innovations may provide an optimal balance by combining strength, adaptability, and resilience to withstand the dynamic pressures of the pelvic area while reducing the risk of complications, but at the moment, evidence is lacking.

## Author Contributions

The idea for the project was conceived by M.A.B. and discussed with E.C.J.C. and S.E.S.K. The protocol and plan were made by M.A.B. Literature searches were performed by M.A.B. and M.G. Data extraction was performed by M.A.B. and M.G. Data analysis was performed by M.A.B. The manuscript was drafted by M.A.B. with editing and input from M.G., P.M.V., E.C.J.C., H.W.R.S. and S.E.S.K.

## Funding

The authors have nothing to report.

## Ethics Statement

The authors have nothing to report.

## Conflicts of Interest

The authors declare no conflicts of interest.

## Data Availability

The data that support the findings of this study are available from the corresponding author upon reasonable request.
